# Case Report: C-arm–assisted peri-hip endoscopic debridement for calcific tendinitis at the deep insertion of the gluteus maximus

**DOI:** 10.3389/fsurg.2026.1805054

**Published:** 2026-06-26

**Authors:** Nan Wang, Gangfeng Hu, Fangqi Xu

**Affiliations:** 1 Department of Traditional Chinese Medicine, The First People's Hospital of Xiaoshan District, Xiaoshan Affiliated Hospital of Wenzhou Medical University, Hangzhou, China; 2 Department of Orthopedic Surgery, The First People's Hospital of Xiaoshan District, Xiaoshan Affiliated Hospital of Wenzhou Medical University, Hangzhou, China

**Keywords:** calcific tendinitis, case report, gluteus maximus, imaging-guided debridement, peri-hip endoscopy

## Abstract

Calcific tendinitis commonly affects the rotator cuff; however, involvement of the deep insertion of the gluteus maximus is uncommon and has been described only in limited reports. Deep-seated hip lesions may be difficult to identify on plain radiographs, potentially resulting in delayed diagnosis. We report the case of a 53-year-old woman who presented with atraumatic lateral hip pain and restricted range of motion for 1 month, that was unresponsive to nonsteroidal anti-inflammatory drugs (NSAIDs). Plain radiograph findings were inconclusive. Magnetic resonance imaging (MRI) showed inflammatory edema surrounding the gluteus maximus insertion, and computed tomography (CT) with three-dimensional (3D) reconstruction identified a calcific deposit. Because of persistent pain, functional limitation, and confirmed deep peri-hip calcification, surgical intervention was selected after discussing nonoperative options. Thus, an imaging-guided peri-hip endoscopic debridement was performed under C-arm fluoroscopic guidance following CT and 3D lesion localization. Typical “toothpaste-like” calcific material extruded from the lesion and was completely removed. Histopathological examination confirmed calcific tendinopathy, and postoperative CT verified complete removal of the lesion. At 6 weeks postoperatively, her pain had resolved, and hip function had markedly improved. This case also highlights an effective, imaging-guided diagnostic workflow for deep peri-hip calcific lesions when radiographic findings are inconclusive. Although a CT-planned, C-arm-assisted approach may facilitate rapid recovery, longer-term follow-up is required to evaluate durability, recurrence, tendon healing, and potential delayed complications.

## 1 Introduction

Hydroxyapatite deposition disease (HADD) is a basic calcium phosphate (BCP)-related crystal deposition disorder. Its primary pathology involves hydroxyapatite crystal deposition in the tendons and adjacent periarticular soft tissues (e.g., bursae and joint capsule), which may induce local inflammation of varying severity (1–3). Most cases have a self-limiting course (1), and clinical manifestations are not specific. At atypical sites or during marked inflammation, HADD may mimic trauma, infection, or neoplasms, leading to misdiagnosis and unnecessary investigations or even invasive procedures (3, 4). Calcific tendinitis surrounding the hip typically occurs near the greater trochanter at the insertion point of the gluteal tendons. However, involvement of the deep insertion of the gluteus maximus at the gluteal tuberosity is infrequently reported and mostly documented as case reports or small case series (5).

Imaging is crucial for diagnosis and differential diagnosis. Although plain radiography is commonly used for initial screening, the deep anatomy of the hip and overlapping osseous structures may render radiographs negative or show only subtle soft tissue density changes (6). Computed tomography (CT) is more sensitive in detecting calcific deposits and enables precise localization, attenuation quantification, and assessment of extent (7). Magnetic resonance imaging (MRI) is particularly useful for demonstrating peritendinous edema and associated intra-articular pathology (6, 7).

Herein, we report a case of calcific tendinitis at the deep insertion of the gluteus maximus in which radiographs were inconclusive, whereas CT and MRI enabled accurate lesion localization. C-arm–assisted hip endoscopic debridement was performed, and histopathological examination confirmed calcific tendinopathy. Postoperative CT confirmed complete removal, and quantitative follow-up demonstrated substantial pain relief and functional improvement. This report aims to demonstrate a practical, integrated imaging-guided approach for diagnosing and surgically treating calcific tendinitis at the deep insertion of the gluteus maximus when radiographs are inconclusive.

## 2 Case description

A 53-year-old woman (height, 158 cm; weight, 70 kg; body mass index, 28.0 kg/m^2^) was admitted with atraumatic lateral left hip pain and limited activity lasting 1 month. Oral nonsteroidal anti-inflammatory drugs (NSAIDs; celecoxib, 200 mg once daily) provided inadequate relief. Other conservative treatment approaches such as physical therapy, image-guided injections, extracorporeal shock wave therapy, and percutaneous needling or lavage were considered. She had a long history of sedentary work and did not consume tobacco or alcohol. Her medical history included bronchial asthma and a cerebral infarction approximately 20 years earlier, with residual left-sided weakness that had largely resolved. She reported no remarkable family medical history.

Physical examination revealed an antalgic gait. The range of motion of the left hip included flexion to 90°, abduction to 15°, and adduction to 10°. Both the flexion–adduction–internal rotation (FADIR) and the flexion–abduction–external rotation (FABER) tests were positive. Substantial tenderness was observed approximately 4 cm inferior to the posterior aspect of the greater trochanter and approximately 1 cm lateral to the femoral axis, corresponding to the surface projection of the deep insertion of the gluteus maximus at the gluteal tuberosity. Distal neurovascular examination findings were normal. Laboratory tests revealed a normal leukocyte count (7.59 × 10^9^/L) and a markedly elevated high-sensitivity C-reactive protein level (hs-CRP, 26.90 mg/L). Other routine preoperative laboratory results were unremarkable.

Hip radiographs demonstrated no definite osseous destruction and revealed only subtle, nonspecific findings (Figures 1A,B). MRI (coronal and sagittal proton density fat-suppressed (PD-FS) sequences) demonstrated peritendinous edema surrounding the gluteus maximus insertion. A focal low-signal area suggested calcific deposition, and no definite intra-articular abnormality was identified (Figures 1C,D).

**Figure 1:**
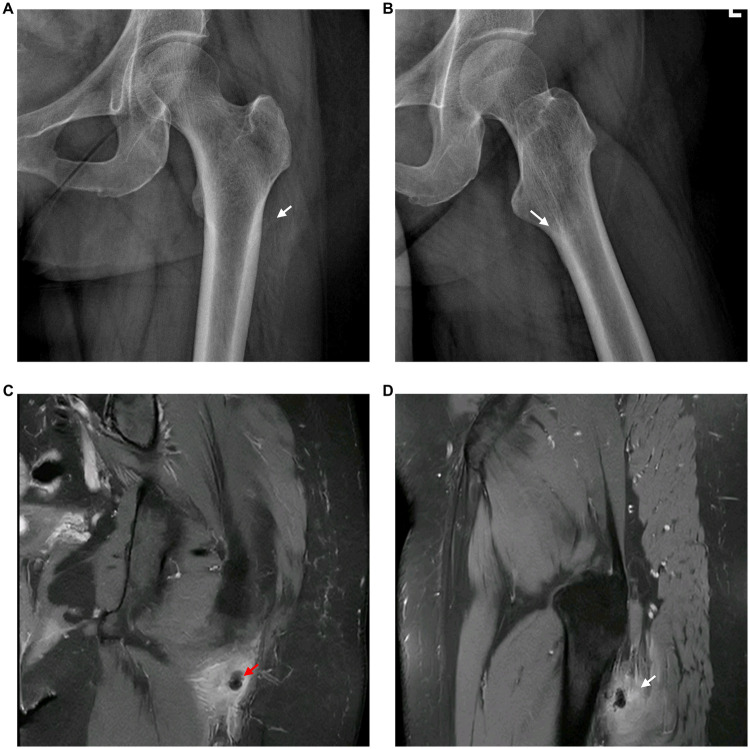
Preoperative radiographs and MRI findings. **(A,B)** Preoperative anteroposterior and lateral radiographs showing a faint mass-like area with slightly increased soft-tissue density (arrow). **(C,D)** Preoperative coronal and sagittal PD-FS MRI showing a low-signal-intensity lesion (red arrow) representing calcific deposition within the gluteus maximus tendon, with surrounding inflammatory edema (white arrow).

CT with 2D and 3D reconstruction revealed a focal high-density deposit at the posterolateral aspect of the femur near the gluteus maximus insertion at the gluteal tuberosity, measuring approximately 1.0 × 2.0 cm. The 3D appearance was flame-shaped. The adjacent cortex remained intact with no evidence of erosion or medullary involvement (Figures 2A–D). CT attenuation was measured on coronal reformats using a circular region of interest (ROI) placed centrally within the deposit while avoiding the margins to minimize partial-volume effects. The mean attenuation (∼200 Hounsfield units (HU)) supported a calcific deposit and facilitated fluoroscopic correlation for portal planning (Figure 2E). Clinical features combined with multimodal imaging supported a diagnosis of calcific tendinitis at the deep insertion of the gluteus maximus. Because of persistent lateral hip pain, peri-trochanteric conditions such as greater trochanteric pain syndrome (GTPS), gluteal tendinopathy, and trochanteric or calcific bursitis were initially considered. However, CT and 3D reconstruction localized the calcific deposit to the deep insertion of the gluteus maximus at the gluteal tuberosity, whereas MRI showed associated peritendinous edema, supporting the diagnosis of insertional calcific tendinitis rather than superficial trochanteric pathology.

**Figure 2:**
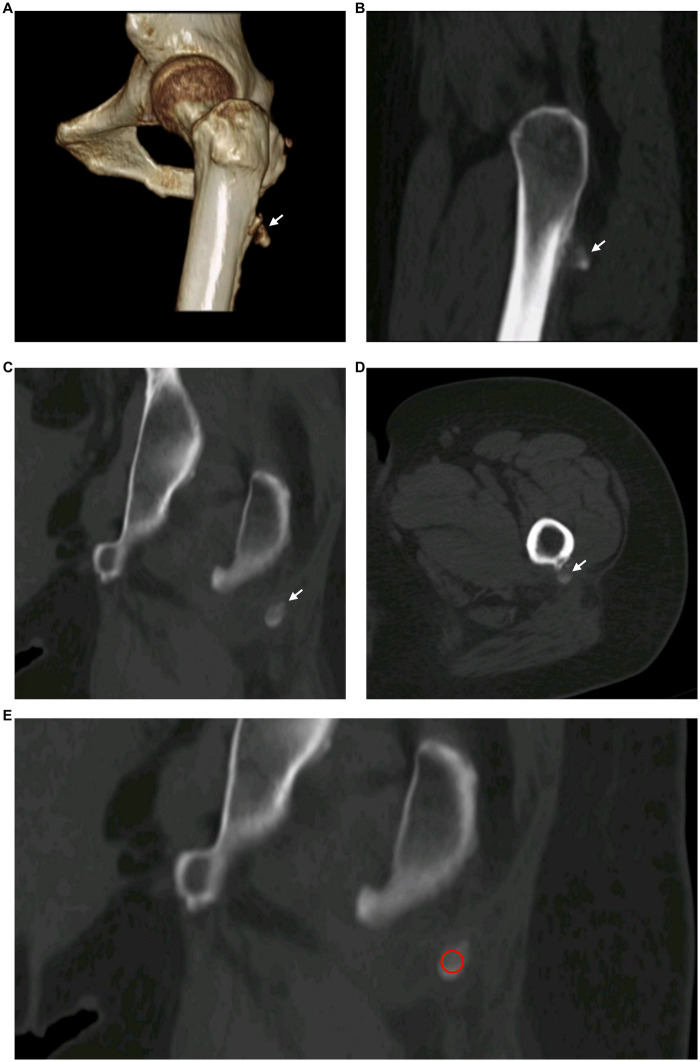
Preoperative CT and 3D reconstruction demonstrating the calcific deposit at the deep insertion of the gluteus maximus and CT attenuation quantification. **(A)** 3D reconstruction showing a focal “flame-shaped” calcific deposit (arrow) at the posterolateral femur near the gluteus maximus insertion (gluteal tuberosity). **(B–D)** Multiplanar reconstructions in the sagittal, coronal, and axial planes demonstrate the same deposit (arrow). **(E)** A circular ROI placed in the central portion of the deposit on the coronal image showing a mean attenuation of approximately 200 HU, supporting a calcific nature. Because HU values may vary with acquisition and reconstruction parameters and ROI placement, attenuation was interpreted as a supportive semiquantitative descriptor rather than a diagnostic threshold.

### 2.1 Timeline

- 4 weeks: Onset of atraumatic lateral hip pain with poor response to NSAIDs.

Admission (day 0): Physical examination demonstrated focal posterolateral hip tenderness. Laboratory investigations revealed a normal white blood cell count and elevated hs-CRP levels.

Imaging (day 0): Plain radiographs were nondiagnostic. MRI showed inflammatory changes surrounding the tendon insertion. 3D- CT localized and quantified the calcific deposit (approximately 200 HU).

Decision-making (day 0): Nonoperative treatment options, including physical therapy and image-guided interventions, were considered. However, because of persistent pain and imaging-confirmed deep peri-hip calcification, surgical intervention was selected.

Surgery (days 0–1): C-arm–assisted peri-hip endoscopic debridement with biopsy.

Postoperative imaging (early): Follow-up CT confirmed complete removal of the calcific deposit.

Follow-up (weeks 1, 2, 4, and 6): The visual analog scale (VAS) score decreased to 0 from week 2 onward. The Harris Hip Score (HHS) improved and remained stable from week 4 onward.

Given the deep lesion location, inconclusive radiographic findings, and definitive localization on CT with 3D reconstruction, image-guided percutaneous interventions were considered technically challenging and diagnostic uncertainty remained. Therefore, peri-hip endoscopic debridement with biopsy was selected to achieve both therapeutic removal and histologic confirmation. Intraoperatively, a straight vascular clamp was inserted through the planned proximal working portal under fluoroscopic guidance, with the clamp tip directed toward the calcific deposit to facilitate accurate portal establishment (Figures 3A,B). Following endoscopic incision, typical “toothpaste-like” calcific material extruded and was thoroughly removed. Repeated irrigation was performed until no noticeable residue was observed (Figures 3C–E). The debrided tissue was subjected to histopathological examination. Postoperatively, celecoxib (200 mg once daily) was administered for 2 weeks. Full weight-bearing was allowed beginning 6 h after surgery. The patient was discharged on postoperative day 3 and was advised to avoid excessive activity for 1 month. She reported good tolerance and adherence to the medication regimen and postoperative instructions, and no intraoperative or early postoperative complications were observed.

**Figure 3:**
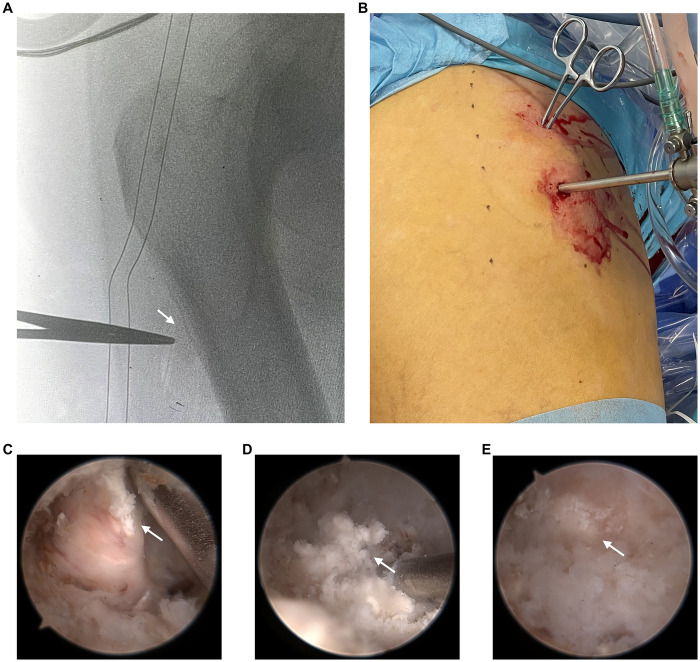
Intraoperative localization and endoscopic debridement. **(A)** Under fluoroscopic guidance, a straight vascular clamp is inserted via the planned proximal portal. The clamp tip points to the calcific deposit at the deep insertion of the gluteus maximus on the posterolateral femur (arrow), assisting precise portal placement. **(B)** The working portal is established, and instruments are introduced. **(C)** The endoscopic incision exposes the lesion area (arrow). **(D)** White “toothpaste-like” calcific material extrudes after incision (arrow). **(E)** After thorough curettage and repeated irrigation, the debrided lesion bed (arrow) shows no obvious residual calcification.

Postoperative CT with 2D and 3D reconstruction confirmed complete removal of the calcific deposit (Figures 4A,B). Hematoxylin and eosin staining demonstrated fibrous connective tissue with calcification and a granulation tissue-like reaction including vascular proliferation, consistent with calcific tendinopathy (Figures 4C,D). Crystal-specific confirmation methods were not performed.

**Figure 4:**
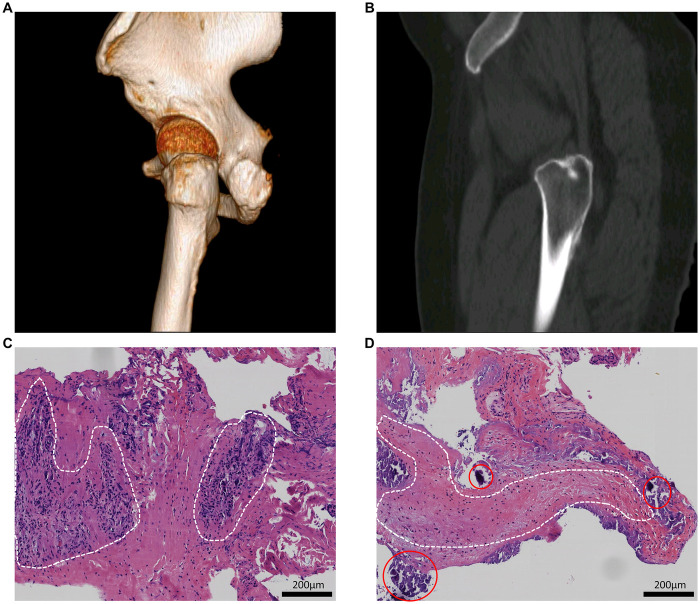
Postoperative CT and pathological evidence. **(A,B)** The calcific deposit previously located at the posterolateral femur near the deep insertion of the gluteus maximus (gluteal tuberosity) has been removed; the bony contour remains intact. **(C)** Granulation tissue-like reaction within the fibrous connective tissue (white dashed outline), characterized by small-vessel proliferation indicating reactive inflammation. **(D)** Fibrocartilaginous change (white dashed outline) with abundant basophilic, amorphous granular deposits consistent with calcification (red circles).

Follow-up at 1, 2, 4, and 6 weeks showed a reduction in VAS pain score from 3 (2 with medication) preoperatively to 1 at 1 week postoperatively and 0 from 2 weeks onward, which was maintained through 6 weeks. The HHS improved from 58.7 preoperatively to 83.0 at 1 week and 93.0 at 2 weeks postoperatively, reaching 100.0 at 4 and 6 weeks. No delayed complications were identified during the 6-week follow-up period; however, a longer-term follow-up is warranted.

## 3 Discussion

Calcific tendinopathy caused by hydroxyapatite crystal deposition is a BCP-related crystal deposition disorder. Clinical symptoms may fluctuate across disease phases, and many cases exhibit a self-limiting course (1–3). However, marked inflammation or atypical anatomical involvement may produce clinical and imaging findings that mimic post-traumatic change, infection, myositis ossificans, or neoplastic conditions, increasing the risk of misdiagnosis and unnecessary investigations or invasive interventions (7–9).

Although calcific tendinitis surrounding the hip is not uncommon, the sites of involvement are variable. Therefore, diagnostic and therapeutic strategies require integration of local anatomy, pain distribution, and imaging characteristics (10). In the present case, the lesion was located at the deep insertion of the gluteus maximus near the gluteal tuberosity, a relatively uncommon site for which the available evidence is largely limited to case reports and small case series (11–14). Clinically, pain was localized to the posterolateral hip and posterior–inferior aspect of the greater trochanter, which may mimic GTPS, gluteal tendinopathy, or lumbar-referred pain. Furthermore, protective muscle spasm and periarticular inflammatory responses around the hip can produce nonspecific positive provocative test findings, potentially directing the diagnostic pathway toward femoroacetabular impingement or intra-articular pathology and thereby increasing the risk of unnecessary intra-articular evaluation and intervention (15).

Differential diagnosis of refractory lateral hip pain should include peri-trochanteric disorders. GTPS is an umbrella term encompassing trochanteric bursitis and hip abductor tendon pathology including gluteus medius or minimus tendinopathy and tendon tears. Imaging findings more commonly demonstrate abductor tendon abnormalities with or without bursal distension rather than isolated primary bursitis (16, 17). Calcium apatite deposition disease may also present as calcific trochanteric bursitis, which is characterized by peri-trochanteric calcification centered within or adjacent to the trochanteric bursa (18). Other causes such as external snapping hip and iliotibial band friction syndrome are predominantly soft-tissue disorders, with MRI often showing thickening of the iliotibial band and/or the anterior band of the gluteus maximus (19). In the present case, CT with 3D reconstruction localized the calcific deposit to the deep insertion of the gluteus maximus at the gluteal tuberosity with corresponding peritendinous edema identified on MRI, favoring insertional calcific tendinitis over GTPS-associated superficial peri-trochanteric disorders.

Imaging plays a central role in the evaluation and differential diagnosis of deep-seated hip lesions. Such lesions may be atypical or remain occult on plain radiographs because of osseous overlap and variations in lesion location or disease phase; similar imaging patterns have been reported in cases involving the gluteus maximus insertion (11–14). CT is the most sensitive modality for detecting calcific deposits and accurately defining their spatial localization, enabling the direct assessment of lesion morphology, extent, and relationship with the adjacent cortex. In cases accompanied by cortical reaction or erosion-like change, CT may also help reduce diagnostic uncertainty with bone tumor-like lesions or heterotopic ossification including myositis ossificans (11, 20). MRI is valuable for assessing peritendinous edema and the extent of tissue involvement and provides complementary information that may assist in excluding infection or neoplastic conditions in diagnostically uncertain cases (7, 8). In selected atypical presentations, bone scintigraphy or single-photon emission computed tomography (SPECT)/CT may demonstrate focal increased uptake and serve as a second-line modality for localization or differential diagnosis (21). In the present case, CT demonstrated a focal calcific deposit at the deep insertion of the gluteus maximus with preservation of the adjacent cortex and no evidence of medullary involvement. In contrast, MRI showed predominantly peritendinous inflammatory edema without a definable intra-articular lesion. Collectively, these findings were consistent with insertional calcific tendinitis and helped exclude intra-articular pathology, infection, heterotopic ossification (including myositis ossificans), and tumor-like lesions.

Treatment should follow a stepwise approach. Most cases can initially be managed with NSAIDs, physical therapy, and activity modification. When necessary, image-guided injection or percutaneous needling/lavage may be considered. In patients with refractory pain, recurrent episodes, or conservative treatment failure, interventional procedures or surgical debridement can provide more definitive symptom relief and promote functional recovery (10). Reported surgical approaches for deep calcific lesions around the hip include open excision and peri-hip endoscopic debridement. In cases involving deep insertion lesions or when localization is challenging, combining peri-hip endoscopy with fluoroscopic guidance may improve lesion localization and procedural precision during the removal of deep peri-hip calcific deposits (5, 21–25). Compared with open surgery, peri-hip endoscopic treatment enables direct visualization and focused debridement with minimal soft tissue disruption while allowing biopsy for histopathological confirmation, thereby strengthening diagnostic certainty (22–25). Although the evidence supporting extracorporeal shock wave therapy and percutaneous lavage is primarily studies of calcific tendinopathy of the shoulder, the same stepwise treatment principles may provide indirect guidance for the management of periarticular calcific disease around the hip (26, 27).

Although most cases of periarticular calcific tendinitis can be managed conservatively, operative management may be appropriate in patients with refractory pain and functional limitation, particularly when lesions are deeply located, radiographs are inconclusive, and CT provides clear lesion localization. In the present case, the calcific deposit was located at the deep insertion of the gluteus maximus, where image-guided percutaneous interventions may be technically challenging and residual diagnostic uncertainty may persist. Peri-hip endoscopic debridement enabled direct visualization and lesion removal while enabling tissue biopsy for histopathological confirmation, thereby providing therapeutic and diagnostic value.

Endoscopic treatment of peri-hip calcific tendinitis, including lesions involving the gluteus maximus insertion, has been reported previously (5, 25). Therefore, we emphasize the integrated imaging-guided workflow rather than procedural novelty. This case highlights the practical value of a CT-planned, fluoroscopy-guided peri-hip endoscopic strategy for radiograph-inconclusive deep peri-hip calcific lesions. Preoperative CT with 3D reconstruction and attenuation assessment guided portal selection and surgical planning, whereas intraoperative C-arm fluoroscopic guidance facilitated coaxial portal placement and limited debridement, helping avoid blind deep exploration and potential injury to adjacent structures. Postoperative CT verification combined with histopathological confirmation further strengthened the diagnostic and therapeutic evidence chain (5, 25).

Histopathology is valuable for confirming the diagnosis in such cases, particularly at atypical sites where excluding more serious pathology is crucial. HADD/BCP-related pathology may demonstrate fibrous tissue proliferation with calcific or mineral deposition and reactive granulation tissue changes, consistent with the tissue response observed during symptomatic phases (2, 3). In the present case, concordant histopathological findings and postoperative CT confirmation of complete lesion removal strengthened the diagnostic credibility of calcific tendinitis occurring at an atypical site.

This report has limitations, notably the short follow-up duration of 6 weeks, which precludes evaluation of recurrence, sustained symptom control, tendon healing or remodeling, and delayed complications. Future studies with longer follow-up periods and larger patient cohorts are needed to confirm the durability and safety of this approach. Nevertheless, in patients with refractory lateral hip pain, deep-seated lesions, and inconclusive radiographic findings, a CT-centered imaging strategy for accurate lesion localization should be considered, with selective MRI used to exclude infection or neoplastic conditions when indicated. In cases of conservative treatment failure or persistent diagnostic uncertainty, minimally invasive hip endoscopic debridement with tissue biopsy may improve diagnostic accuracy while facilitating symptom relief and functional recovery.

In summary, this case highlights calcific tendinitis at the deep insertion of the gluteus maximus as an occult cause of refractory lateral hip pain. Plain radiographs may fail to detect the lesion, and CT and MRI are important for accurate localization and differential diagnosis. In patients with clearly defined lesions and unsuccessful conservative management, a CT-planned, C-arm–assisted peri-hip endoscopic approach may facilitate adequate lesion removal through a minimally invasive technique while achieving rapid symptomatic improvement. Long-term outcomes, including recurrence and delayed complications, remain to be determined.

## 4 Patient perspective

After symptom onset, pain on the lateral aspect of my left hip made walking and daily activities difficult, and the lack of relief with medication increased my anxiety. After the minimally invasive procedure, my pain improved quickly, and I was able to return to normal daily activities. I felt relieved that the cause of my symptoms was clearly identified and treated.

## Data Availability

The raw data supporting the conclusions of this article will be made available by the authors, without undue reservation.
